# CAGEs are Golgi‐localized GT31 enzymes involved in cellulose biosynthesis in Arabidopsis

**DOI:** 10.1111/tpj.15734

**Published:** 2022-03-31

**Authors:** Pieter Nibbering, Romain Castilleux, Gunnar Wingsle, Totte Niittylä

**Affiliations:** ^1^ Department of Forest Genetics and Plant Physiology Umeå Plant Science Centre, Swedish University of Agricultural Sciences 901 83 Umeå Sweden

**Keywords:** Arabidopsis, cell wall, cellulose, arabinogalactan protein, Golgi, glycosyltransferase family 31

## Abstract

Cellulose is the main structural component in the plant cell walls. We show that two glycosyltransferase family 31 (GT31) enzymes of *Arabidopsis thaliana*, here named cellulose synthesis associated glycosyltransferases 1 and 2 (CAGE1 and 2), influence both primary and secondary cell wall cellulose biosynthesis. *cage1cage2* mutants show primary cell wall defects manifesting as impaired growth and cell expansion in seedlings and etiolated hypocotyls, along with secondary cell wall defects, apparent as collapsed xylem vessels and reduced xylem wall thickness in the inflorescence stem. Single and double *cage* mutants also show increased sensitivity to the cellulose biosynthesis inhibitor isoxaben. The *cage1cage2* phenotypes were associated with an approximately 30% reduction in cellulose content, an approximately 50% reduction in secondary cell wall CELLULOSE SYNTHASE (CESA) protein levels in stems and reduced cellulose biosynthesis rate in seedlings. *CESA* transcript levels were not significantly altered in *cage1cage2* mutants, suggesting that the reduction in CESA levels was caused by a post‐transcriptional mechanism. Both CAGE1 and 2 localize to the Golgi apparatus and are predicted to synthesize β‐1,3‐galactans on arabinogalactan proteins. In line with this, the *cage1cage2* mutants exhibit reduced levels of β‐Yariv binding to arabinogalactan protein linked β‐1,3‐galactan. This leads us to hypothesize that defects in arabinogalactan biosynthesis underlie the cellulose deficiency of the mutants.

## Significance Statement

Cellulose, the main component of plant cell walls, is synthesized at the plasma membrane by a multiprotein complex with cellulose synthase enzymes at its core. Here, we show that two Golgi localized glycosyltransferase enzymes are indirectly involved in cellulose biosynthesis, likely by acting on the synthesis of glycans on arabinogalactan proteins.

## INTRODUCTION

Plant cell walls are necessary for plant growth and development, as well as one of the most bountiful sustainable raw materials. The main structural component in plant cell walls is cellulose, which consists of crystalline β‐1,4‐glucan chain microfibrils. Plant cell expansion driven growth and the final cell dimensions are dictated by turgor‐driven anisotropic extension of the primary cell wall, which largely depends on cellulose microfibril (CMF) orientation and CMF interactions with the cell wall matrix (Cosgrove, 2005). CMFs also contribute to the mechanical strength of secondary cell walls, which are synthesized in specific cell types, such as xylem vessels and fibers, after cell expansion (Kumar et al., 2016). Mutations affecting the biosynthesis of primary cell wall cellulose typically manifest as decreased cell expansion and growth defects in seedlings (Baskin et al., 1992; Persson et al., 2007), whereas a decrease in secondary cell wall cellulose levels can stunt growth and weaken stems, as well as result in thinner fiber cell walls and collapsed xylem vessels (Taylor et al., 2003; Turner & Somerville, 1997).

The β‐1,4‐glucan chains of CMFs are synthesized at the plasma membrane by cellulose synthases (CESAs). In *Arabidopsis thaliana* (Arabidopsis), primary cell wall cellulose is synthesized by CESA1‐, CESA3‐ and CESA6‐related proteins, whereas CESA4, CESA7 and CESA8 synthesize cellulose for the secondary cell walls (Desprez et al., 2007; Persson et al., 2007; Taylor et al., 2003). CESAs assemble within the catalytic core of the cellulose synthase complex (CSC) in the Golgi apparatus, from where they are transported to the plasma membrane (Crowell et al., 2009). Besides CESAs, several other proteins are involved in the *in vivo* biosynthesis of cellulose by supporting the assembly (Vain et al., 2014; Zhang et al., 2016) and trafficking of CSCs along cortical microtubules (Bringmann et al., 2012; Gu et al., 2010). A comprehensive account of CSC‐associated proteins can be found in reviews by Polko & Kieber (2019) and Lampugnani et al. (2019). In addition to the core CSC‐associated components, there is experimental evidence implicating a role for arabinogalactan proteins (AGPs) in cellulose biosynthesis (Seifert, 2018).

AGPs in plants are subdivided into the classical AGPs, lysine‐rich AGPs, AG peptides, fasciclin‐like AGPs (FLAs) and other chimeric AGPs (Showalter et al., 2010). Of the AGPs characterized until now, only members of the FLA family have been linked to cellulose biosynthesis. Arabidopsis contains 21 FLAs, which are further classified into four subgroups (labeled A, B, C and D) based on their amino acid sequence and domain structures (Johnson et al., 2003). The evidence for FLA involvement in cellulose biosynthesis comes from studies of Arabidopsis and *Populus* mutants. For example, *fla4* (also known as *salt overly sensitive 5*) mutants exhibit root cell expansion defects when grown on media with a high NaCl content (Shi et al., 2003). The cell expansion phenotype was associated with conditional cellulose biosynthesis defects in primary cell walls; more specifically, *fla4* seedlings incorporated less ^14^C‐glucose into cellulose under NaCl stress (Basu et al., 2016). FLAs have also been linked to secondary cell wall cellulose biosynthesis, with MacMillan et al. (2010) demonstrating that the simultaneous mutation of *FLA11* and *FLA12* results in reduced cellulose content in the inflorescence stem of Arabidopsis. Also, *Populus* lines with RNAi‐induced suppression of AtFLA11/12 orthologs displayed reduced cellulose levels in stems (Wang et al., 2015a). Recent characterization of Arabidopsis *FLA11* and *FLA12* overexpression and mutant lines supported a function in response to mechanical stress during secondary cell wall biosynthesis for these FLAs (Ma et al., 2022). A direct FLA role in cellulose biosynthesis was suggested by the observation that orthologs of Arabidopsis FLA7 and FLA11 co‐immunoprecipitated with secondary cell wall CESAs from developing xylem of *Populus deltoides* × *trichocarpa* and cotton fiber extracts (Li et al., 2016; Song et al., 2010). However, it is not known how FLAs influence cellulose biosynthesis, although structural roles in cell wall matrix assembly, direct interaction with CSCs and receptor‐mediated signaling mechanisms have been proposed (Seifert, 2018; Tan et al., 2013; Xue et al., 2017). In general, it has been challenging to establish mechanistic relationships between AGP functionality and plant cell wall assembly. The glycans attached to the AGP protein core are assumed to be important for functionality, and the characterization of the enzymes responsible for glycan biosynthesis may provide insight into AGP function and their functional redundancy (Knoch et al., 2014).

AGP glycans consist of a β‐1,3‐galactan backbone with the β‐1,6‐linked side chains including galactose, arabinose, glucuronic acid, rhamnose and fucose (Kitazawa et al., 2013; Tan et al., 2010; Tryfona et al., 2010; Tryfona et al., 2012). The *O*‐glycosylation of AGPs starts in the ER, where proline hydroxylation by proly‐4‐hydroxylases occurs, after which the first galactose may be attached to hydroxyproline (Hieta & Myllyharju, 2002; Hijazi et al., 2014; Rose & Lee, 2010). After exiting the ER, glycosylation continues in the Golgi apparatus, where a group of glycosyl transferases (GTs) synthesize arabinogalactan. One of the key GT families involved in AGP glycosylation is the glycosyl transferase 31 (GT31) family, which includes enzymes that participate in the synthesis of the β‐1,3‐galactan backbone. The Arabidopsis GT31 family consists of 33 members, of which 20 were initially suggested to be involved in the glycosylation of AGPs based on the presence of a putative β1,3‐galactosyltransferase domain (Qu et al., 2008). Phylogenetic analyses have subsequently subdivided these twenty GT31s into six clades, with the assignments based on biochemical activity predictions from sequence similarity to GT31s characterized from *Homo sapiens* (Qu et al., 2008). Clades I and II were predicted to have β1,3‐galactosyl transferase activity, clade III was predicted to have β1,3‐*N*‐acetylglucosaminyltransferase activity, clade IV was predicted to have β1,3‐galactosyl transferase activity for *N*‐glycosylation, and clades V and VI were predicted to have hydroxyproline *O*‐galactosyltransferase activity (Qu et al., 2008). Some of these predictions have been experimentally validated; for example, GALACTOSYLTRANSFERASE1 (GALT1) from clade V was found to be a β‐1,3‐galactosyltransferase essential for the attachment of β1,3‐galactose residues to *N*‐glycans (Strasser et al., 2007). Moreover, GALT2‐GALT6 (clades V and VI) and hydroxyproline *O*‐galac‐tosyltransferases (HPGTs, clade III) have been shown to be hydroxyproline *O*‐galactosyltransferases (Basu et al., 2013; Basu et al., 2015a; Basu et al., 2015b; Ogawa‐Ohnishi & Matsubayashi, 2015). As such, *galt2‐galt6* mutants displayed multiple growth defects, including reduced root hair growth, as well as reduced seed coat mucilage and seed set, along with accelerated leaf senescence and reduced root growth under salt stress (Basu et al., 2015a; Basu et al., 2015b). Furthermore, triple *hpgt* mutants (*hpgt1,2,3*) with only 10% of wild‐type (WT) HPGT activity remaining, exhibited a reduced overall growth rate, but had longer lateral roots and root hairs, as well as increased radial expansion of cells in the root tip (Ogawa‐Ohnishi & Matsubayashi, 2015). The *A. thaliana* GT31 enzymes from clade IV have not been characterized, but cotton orthologs suggest that clade IV possesses β‐1,3‐galactosyltransferase activity (Qin et al., 2017). The enzyme GALT31A (clade II) was initially characterized to have β‐1,6‐galactosyltransferase activity (Geshi et al., 2013), although a recent study indicated that it is actually a β‐1,3‐galactosyltransferase (Ruprecht et al., 2020). Mutations in Arabidopsis *GALT31A* were found to arrest embryonic development at the globular stage, which indicates that this protein and the β‐1,3‐galactan linkages it catalyzes are crucial in plants (Geshi et al., 2013). A close clade II homolog of GT31A called KAONASHI4 (KNS4) was also shown to be a β‐1,3‐galactosyltransferase, with *kns4* mutants displaying reduced fertility, shorter seed pods and reduced seed set as a result of defects in pollen exine development (Suzuki et al., 2017). Thus, the biosynthetic activity has been established for 11 out of the 20 putative AGP glycosylating GT31s. Moreover, the characterization of corresponding Arabidopsis mutants has established that GT31s function in a wide variety of growth and developmental processes in plants. However, the *in vivo* targets of these enzymes have not yet been established; therefore, the link between GT31 activity and plant phenotype often remains unclear. Here, we present results on two previously uncharacterized *GT31* genes from Arabidopsis, and describe their involvement in primary and secondary cell wall cellulose biosynthesis.

## RESULTS

### *CAGE1* and *CAGE2* mutants display primary and secondary cell wall defects

The analysis of co‐expressed genes has proven effective for identifying functionally related cell wall biosynthesis genes in Arabidopsis (Aoki et al., 2007; Mutwil et al., 2009; Ruprecht et al., 2011). In an effort to investigate the role of β1,3‐galactans in plant cell walls, we recently identified two Golgi‐localized exo‐β1,3‐galactosidases involved in cell expansion and root growth in Arabidopsis through their involvement in AGP glycan synthesis in the Golgi (Nibbering et al., 2020). To identify candidate enzymes participating in β1,3‐galactosidase substrate synthesis *in vivo*, we searched for co‐expressed putative β1,3‐galactosyltransferase genes using the publicly available Arabidopsis co‐expression tools (https://atted.jp and https://genevestigator.com). This resulted in the identification of co‐expressed candidates from the GT31 family clade I, namely, AT1G05170 and its close sequence homolog AT4G26940 (Table S1). The genes were named cellulose synthesis associated glycosyltransferases 1 and 2 (*CAGE1* and *2*) based on the mutant phenotype described below. Both *CAGE1* and *CAGE2* are expressed throughout the Arabidopsis plant, with the bottom internodes of the inflorescence stem showing the highest expression (http://bar.utoronto.ca/eplant). We also noted that the co‐expressed gene list included several known cellulose biosynthesis associated genes, including *CESA*s, *STELLO1* and *2*, *CSI1*, and *POM1*, as well as four *FLA*s (Table S1). The predicted catalytic region of CAGE1 and CAGE2 show 88% amino acid sequence similarity, and 70–71% similarity with the phylogenetically related enzymes with established β1,3‐galactosyltransferase activity (Figure S1).

**Figure 1:**
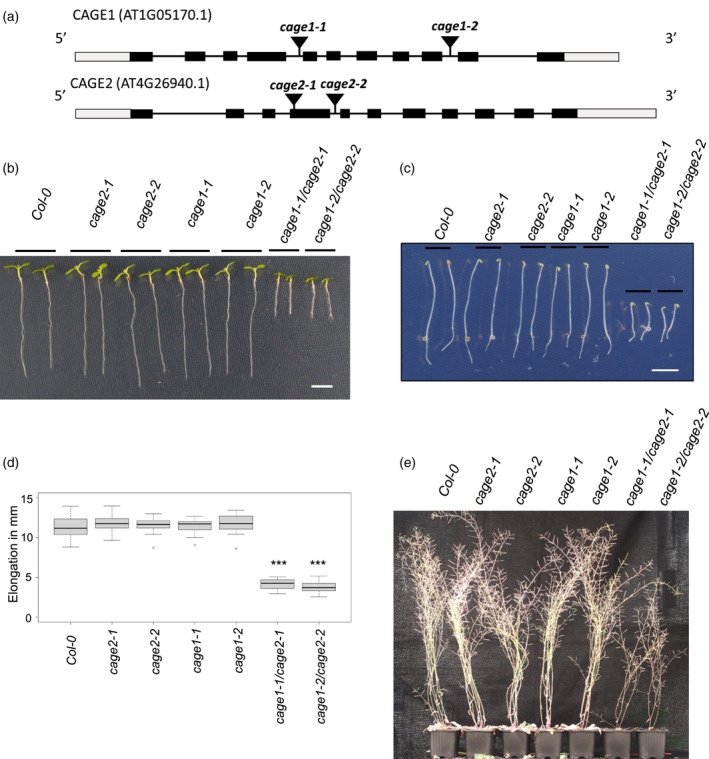
Characterization of the *cage1* and *cage2* T‐DNA insertion lines. (a) Schematic diagram of the *CAGE1* and *CAGE2* gene structure along with the T‐DNA insertion sites. The gray and black boxes indicate untranslated and translated regions, respectively. The lines indicate introns. (b) Seven‐day‐old *Col‐0* and *cage* seedlings grown on ½ MS agar plates (scale bar = 5 mm). (c) Four‐day‐old etiolated *Col‐0* and *cage* seedlings grown on ½ MS agar plates in the dark (scale bar = 5 mm). (d) Hypocotyl length of four‐day‐old etiolated *Col‐0* and *cage* seedlings. In the boxplot, dark horizontal lines represent the median, the two gray boxes denote the 25th and 75th percentiles, whiskers denote the 1.5 interquartile range limits, and the dots are outliers. ***P < 0.001 (unpaired *t*‐test, *n* = 25–30 biological replicates). (e) Ten‐week‐old senescing *Col‐0* and *cage* lines. [Colour figure can be viewed at wileyonlinelibrary.com]

**Figure 2:**
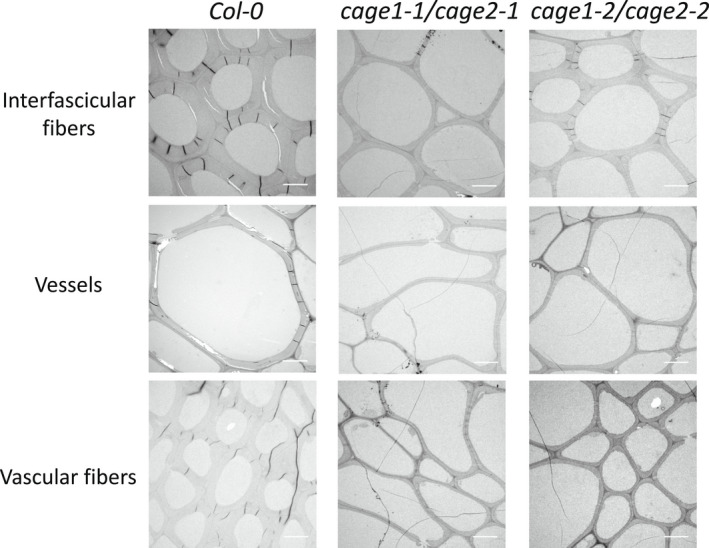
Transmission electron microscope images of interfascicular fibers, vessels and vascular fibers. Images are obtained from cross‐sections of 10‐week‐old inflorescence stems of *Col‐0*, *cage1‐1/cage2‐1* and *cage1‐2/cage2‐2*. Scale bar = 5 μm.

To investigate the function of *CAGE1* and *CAGE2*, we obtained T‐DNA insertion mutants for both genes (Figure 1a). Homozygous single *CAGE1* or *CAGE2* T‐DNA insertion lines did not show obvious phenotypical differences relative to WT plants when grown under controlled conditions, but double *cage1cage2* mutants demonstrated clear growth defects. Allelic *cage1‐1/cage2‐1* and *cage1‐2/cage2‐2* seedlings both exhibited shortened primary roots and etiolated hypocotyls (Figure 1b,c). Reverse transcriptase‐polymerase chain reaction (RT‐PCR) analysis using primers flanking the intron T‐DNA insertion sites in *CAGE1* and *CAGE2* indicated that the alleles are loss of function mutants (Figure S2). Quantification of the etiolated hypocotyl length confirmed a significant reduction in both double mutant lines relative to WT seedlings (Figure 1d). Because hypocotyl elongation is primarily driven by cell expansion rather than cell division, this observation supports that CAGE1 and CAGE2 are involved in primary cell wall biosynthesis and cell expansion. At maturity, the inflorescences of 10‐week‐old *cage1‐1/cage2‐1* and *cage1‐2/cage2‐2* mutants were clearly stunted, indicating that both CAGE1 and CAGE2 also function after the seedling stage (Figure 1e). Cross‐sections of the 10‐week‐old inflorescence stems of the *cage1cage2* lines revealed reduced fiber cell wall thickness and the occasional collapse of xylem vessels (Figures S3 and S4). Moreover, transmission electron microscope (TEM) analysis of the cross‐sections confirmed thinner secondary cell walls in the vessels, vascular fibers and interfascicular fibers of the *cage1cage2* mutants relative to WT plants (Figure 2). No difference was observed in the TEM images of *cage1* or *cage2* single mutant stem cross‐sections (Figure S5). Quantification of the cell wall thickness of interfascicular fibers confirmed these results (Figure S6). To establish a causal link between the phenotypes and T‐DNA insertions, the *cage1‐1/cage2‐1* mutant was transformed with either *CAGE1‐YFP* or *CAGE2‐YFP* under the control of a native promoter. Both constructs complemented the etiolated hypocotyl phenotype (Figure S7a,b) and the stunted growth in soil‐grown plants (Figure S8).

### *cage1cage2* mutants demonstrate defective cellulose biosynthesis

**Figure 3:**
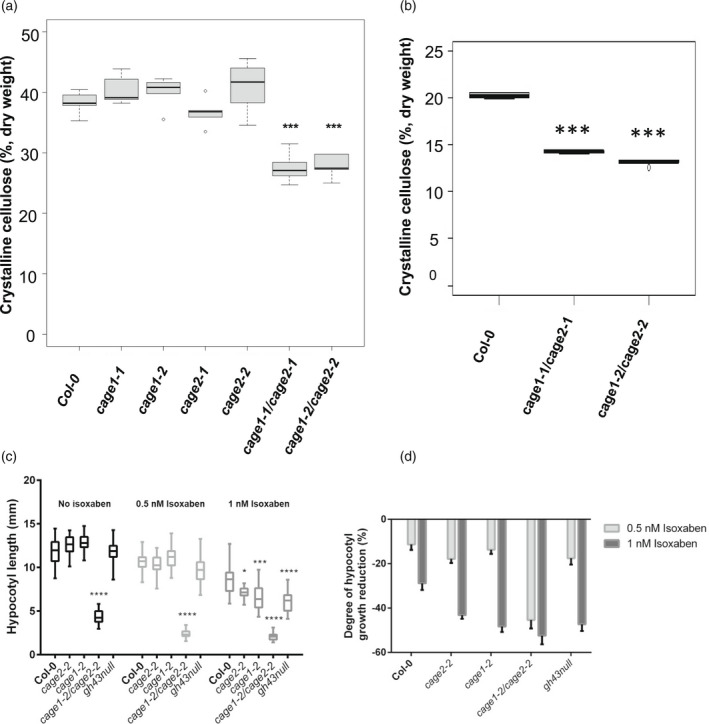
Crystalline cellulose content in 10‐week‐old inflorescence stems and 4‐day‐old dark grown hypocotyls and the effect of isoxaben on hypocotyl etiolation in *Col‐0*, *cage1*, *cage2* and *cage1cage2* lines. (a) Crystalline cellulose content (% of dry weight) in 10‐week‐old inflorescence stems. In the boxplot, dark horizontal lines represent the median, the two gray boxes denote the 25th and 75th percentiles, whiskers denote the 1.5 interquartile range limits, and the dots are outliers. ***P < 0.001 (unpaired *t*‐test, *n* = 6 biological replicates). (b) Crystalline cellulose content (% of dry weight) in 4‐day‐old dark grown hypocotyls. In the boxplot, dark horizontal lines represent the median, the two gray boxes denote the 25th and 75th percentiles, whiskers denote the 1.5 interquartile range limits, and the dots are outliers. ***P < 0.001 (unpaired *t*‐test, *n* = 5 biological replicates of pooled seedlings). (c) Length of 4‐day‐old hypocotyls grown in the dark on ½ MS medium with or without isoxaben. Populations do not all follow a normal distribution (d'Agostino test at α = 5%, *n* ≥ 26). For a given isoxaben concentration, a comparison was made with Col‐0: Kruskal–Wallis test at α = 5%. *P < 0.05; **P < 0.01; ***P < 0.001; ****P < 10^−4^. (d) Percentage decrease of hypocotyl length upon isoxaben treatment. Error bars indicate the SEM.

To investigate the cause of the decreased cell expansion and wall defects, we analyzed the cell wall composition of the mutants. The single *cage1* and *cage2* mutants did not differ significantly from WT lines in terms of cellulose content, whereas the *cage1cage2* lines showed an approximately 30% decrease in cellulose content relative to WT lines in both 10‐week‐old inflorescence stems and 4‐day‐old dark‐grown hypocotyls (Figure 3a,b). The cellulose content was fully restored in the stems of the *CAGE1‐YFP* or *CAGE2‐YFP* complemented *cage1cage2* lines, which confirms a causal link between both CAGE1 and CAGE2 and cellulose synthesis (Figure S9). The mass fraction of cell wall matrix sugars and lignin was increased in the mutants, likely reflecting the decrease in cellulose (Table 1 and Figure S10). Analysis of cell wall matrix monosugar composition in the *cage1cage2* inflorescence stems showed higher levels of rhamnose, xylose, mannose, galacturonic acid and methylated glucuronic acid compared to the WT stems (Table 1). We also noted that the amount of galactose did not differ between the mutant and WT lines, which demonstrates that CAGE1 and CAGE2 are not major contributors to the cell wall galactose pool (Table 1).

**Table 1:** Hemicellulosic/pectic sugar content of 10‐week‐old stems from *Col‐0* and *cage* lines (%, dry weight)

	*Col‐0*	*cage2‐1*	*cage2‐2*	*cage1‐1*	*cage1‐2*	*cage1‐1/cage2‐1*	*cage1‐2/cage2‐2*
Arabinose	1.25 ± 0.03	1.30 ± 0.03	1.36 ± 0.05	1.25 ± 0.02	1.24 ± 0.02	1.25 ± 0.03	1.33 ± 0.03*
Rhamnose	1.32 ± 0.04	1.42 ± 0.03	1.45 ± 0.08	1.35 ± 0.03	1.33 ± 0.02	1.46 ± 0.04*	1.59 ± 0.05**
Fucose	0.92 ± 0.02	0.98 ± 0.02*	0.95 ± 0.02	0.94 ± 0.01	0.90 ± 0.02	0.95 ± 0.03	0.95 ± 0.02
Xylose	15.09 ± 0.54	14.78 ± 0.46	15.92 ± 1.04	15.48 ± 0.64	15.34 ± 0.40	17.21 ± 0.48*	18.99 ± 0.65**
Mannose	1.83 ± 0.08	1.90 ± 0.05	1.89 ± 0.12	1.78 ± 0.07	1.92 ± 0.06	2.06 ± 0.05*	2.14 ± 0.07*
Galactose	1.74 ± 0.03	1.78 ± 0.04	1.83 ± 0.06	1.72 ± 0.01	1.70 ± 0.03	1.77 ± 0.04	1.81 ± 0.04
Galacturonic acid	3.94 ± 0.17	4.38 ± 0.10	4.57 ± 0.28	3.98 ± 0.07	4.16 ± 0.09	4.84 ± 0.24*	5.31 ± 0.20***
Glucose	4.76 ± 0.15	5.28 ± 0.36	5.17 ± 0.18	4.29 ± 0.15	4.71 ± 0.32	5.02 ± 0.32	4.46 ± 0.20
Glucuronic acid	1.27 ± 0.04	1.32 ± 0.02	1.32 ± 0.05	1.28 ± 0.02	1.30 ± 0.03	1.27 ± 0.03	1.35 ± 0.02
Methyl Glucuronic acid	1.68 ± 0.08	1.77 ± 0.04	1.75 ± 0.09	1.68 ± 0.06	1.75 ± 0.05	2.09 ± 0.05**	2.18 ± 0.05***
Matrix polysaccharide total	33.80 ± 1.10	34.91 ± 0.89	36.22 ± 1.83	33.76 ± 0.82	34.36 ± 0.85	37.90 ± 1.07*	40.09 ± 1.05**

Alcohol insoluble residue (AIR1) and starch (AIR2) were removed from the cell wall material. The hemicellulosic/pectic sugars were released by methanolysis. Trimethylsilyl (TMS) derivatised signals were obtained via GC‐MS and quantified in accordance with known sugar standards.

Unpaired *t*‐test, *P < 0.05, **P < 0.01, ***P < 0.001 (*n* = 6 biological replicates).

Primary cell wall cellulose biosynthesis associated mutants display increased sensitivity to the cellulose synthesis inhibitor isoxaben (Desprez et al., 2002; McFarlane et al., 2014). The single *cage1* and *cage2* mutants and the *cage1cage2* also displayed increased sensitivity of dark‐grown hypocotyl elongation to isoxaben, similar to cellulose biosynthesis defect mutants (Figure 3c,d). Only one mutant allele is shown here, although similar results were observed with the other mutant allele (data not shown). Furthermore, the *gh43null*, impaired in the two exo‐β1,3‐galactosidases co‐expressed with the *CAGE1* and *2*, also displayed increased sensitivity to isoxaben (Figure 3c,d). The *cage1* and *cage2* single mutants and the *gh43null* exhibit no obvious growth or cell wall chemistry defects that could lead to a secondary cellulose biosynthesis defect (Table 1 and Nibbering et al., 2020). Thus, the increased isoxaben sensitivity provides evidence that CAGE1, CAGE2 and also GH43s participate in a cellulose biosynthesis associated process.

**Figure 4:**
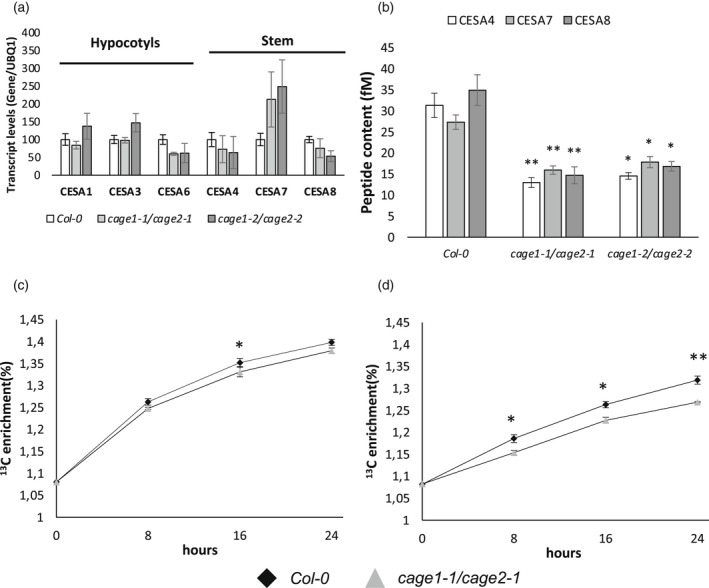
*CESA* transcript, CESA protein levels and cellulose biosynthesis rates measured in *Col‐0* and *cage1cage2* plants. (a) Expression levels of *CESA1*, *3* and *6* in 4‐day‐old etiolated seedlings and *CESA4*, *7* and *8* in the inflorescence stems analyzed by RT‐qPCR (mean ± SE, *n* = 3 biological replicates, UBQ1 = AT3G52590). (b) Secondary cell wall CESA peptide levels from the first 10 cm of 20‐cm long inflorescence stems from *Col‐0* and the *cage1cage2* mutants. Significance is calculated with a two‐sided Student's unpaired *t*‐test. *P ≤ 0.05, **P ≤ 0.01 (mean ± SE, *n* = 4 biological replicates). (c) Total ^13^C enrichment in 8‐day‐old *Col‐0* and *cage1cage2* seedlings after ^13^C‐glucose supply. (d) ^13^C enrichment in crystalline cellulose in 8‐day‐old *Col‐0* and *cage1cage2* seedlings. ^13^C enrichment was determined with an elemental analyzer‐isotope ratio mass spectrometer and is shown as a percentage of total carbon. The data represents three biological replicates (mean ± SD) of approximately 1000 pooled seedlings per time point. Significance is calculated with a two‐sided Student's unpaired *t*‐test. *P ≤ 0.05, **P ≤0.01.

To investigate the cause of the cellulose defect in *cage1cage2* mutants, we analyzed the expression levels of primary cell wall *CESA1*, *CESA3* and *CESA6* in 4‐day‐old dark‐grown hypocotyls, along with the expression of secondary cell wall *CESA4*, *CESA7* and *CESA8* in the inflorescence stem, using a quantitative PCR (qPCR). A tendency of increased *CESA7* transcript levels was observed in the *cage1cage2* lines, but none of the primary or secondary cell wall *CESA* transcript level changes were statistically significant (Figure 4a). Thus, the cellulose defect is not caused by transcriptional deficiency. To investigate CESA protein levels, secondary cell wall CESA4, 7 and 8 were analyzed using quantitative mass spectrometry and isotope‐labeled standard peptides in accordance with a previously reported method (Zhang et al., 2018). This assay revealed significant reductions (approximately 50%) in secondary cell wall CESA protein levels in the *cage1cage2* mutants (Figure 4b). This conclusion was also supported by reduced band intensity of CESA4, 7 and 8 in *cage1cage2* compared to WT on western blots probed with CESA isoform specific antibodies (Figure S11).

Because we do not have a standard peptide‐based mass spectrometry method for primary wall CESA quantification, we instead investigated the rate of primary cell wall cellulose biosynthesis by comparing the rates at which growth media‐supplied ^13^C‐glucose was incorporated into cellulose in *cage1cage2* and WT seedlings. A time course experiment revealed a significant reduction in the amount of ^13^C in the crystalline cellulose fraction of *cage1cage2* cell walls at all time points compared to WT, whereas total ^13^C uptake was significantly lower in *cage1cage2* mutants relative to the WT line only at the 16‐h time point (Figure 4c,d). Combined, the seedling and stem results suggest that the reduced cellulose content observed in *cage1cage2* mutants is caused by a post‐transcriptional mechanism that reduces CESA levels and, subsequently, the rate of cellulose biosynthesis.

### CAGE1‐YFP and CAGE2‐YFP are Golgi‐localized

**Figure 5:**
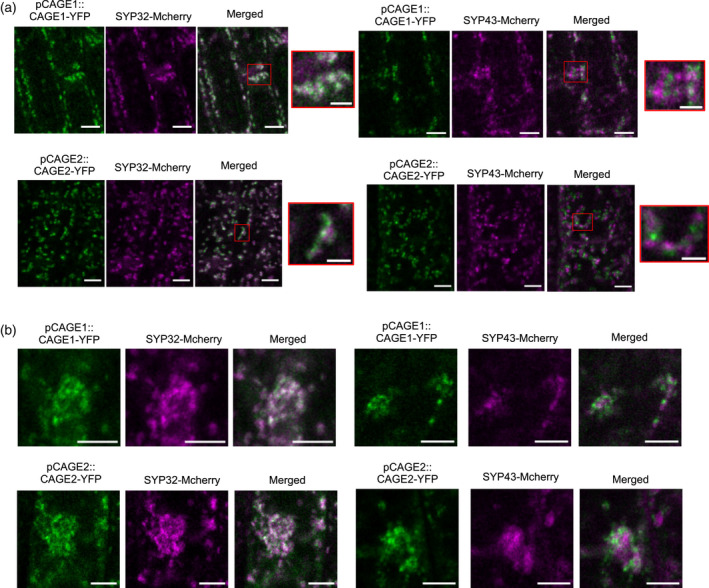
Co‐localization of CAGE1‐YFP and CAGE2‐YFP with mCherry Golgi markers. (a) Confocal laser scanning microscope images of root epidermis cells in Arabidopsis seedlings stably expressing pCAGE1:CAGE1‐YFP or pCAGE2:CAGE2‐YFP and the *cis*‐Golgi marker SYP32‐mCherry or the TGN marker SYP43‐mCherry. Images were captured with a LSM880 confocal microscope (Carl Zeiss, Jena, Germany). Scale bars = 5 μm. (2 μm for zoomed image). (b) Confocal laser scanning microscope images of root epidermis cells in Arabidopsis seedlings stably expressing pCAGE1:CAGE1‐YFP or pCAGE2:CAGE2‐YFP and the *cis*‐Golgi marker SYP32‐mCherry or the TGN marker SYP43‐mCherry after treatment with 25 μm brefeldin A for 30 min. Images were captured with a LSM880 confocal microscope (Carl Zeiss). Scale bars = 5 μm. [Colour figure can be viewed at wileyonlinelibrary.com]

We next established the subcellular localisation of CAGE1 and CAGE2 using the *CAGE1‐YFP* and *CAGE2‐YFP* lines. In the epidermal cells of the root elongation zone of 4‐day‐old seedlings, both CAGE1‐YFP and CAGE2‐YFP appeared to be localized to the Golgi apparatus (Figure 5). To validate the Golgi localisation, the lines were crossed with the *cis*‐Golgi marker SYP32‐mCherry and the *trans*‐Golgi network (TGN) marker SYP43‐mCherry (Geldner et al., 2009; Uemura et al., 2004). Both CAGE1‐YFP and CAGE2‐YFP co‐localized with the Golgi markers, and showed stronger overlap with SYP32‐mCherry than with SYP43‐mCherry (Figure 5a). It was difficult to quantify the extent of co‐localization because of the weakness of the YFP signal. Therefore, to confirm that the CAGE1/2‐YFP proteins were localized to the Golgi rather than the TGN, the lines were treated with brefeldin A for 30 min. Brefeldin A causes the fusion of early Golgi cisternae and the endoplasmic reticulum, whereas Golgi cisternae facing the trans side of the Golgi and TGN are dispersed and accumulate in brefeldin A induced compartments (Nebenfuhr et al., 2002). After the brefeldin A treatment, both CAGE1‐YFP and CAGE2‐YFP co‐localized with SYP32‐mCherry, which confirms a *cis‐*Golgi and/or medial‐Golgi localisation (Figure 5b).

### *cage1cage2* mutants show decreased levels of AGP‐linked β‐1,3 galactan

**Figure 6:**
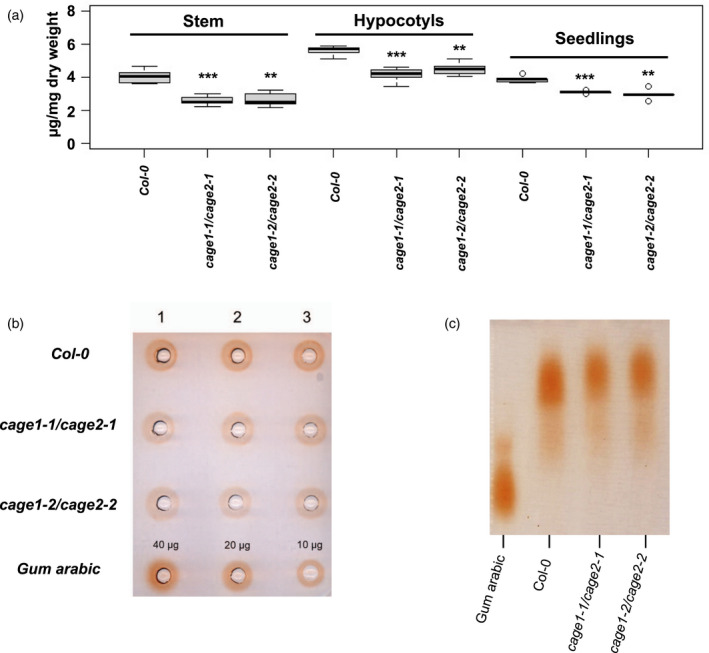
β‐Yariv quantification and characterization of AGPs from Col‐0 and *CAGE1CAGE2* lines. (a) β‐Yariv precipitated AGP quantification from the bottom 10 cm of inflorescence stem, 4‐day‐old etiolated hypocotyls and 7‐day‐old seedlings of Col‐0 and *CAGE1CAGE2* lines. β‐Yariv binding to Gum Arabic was used as standard. The boxplot's dark horizontal lines represent the median, the two gray boxes the 25th and 75th percentile, whiskers the 1.5 interquartile limits, and the dots are outliers. **P < 0.01, ***P < 0.001 (unpaired *t*‐test, *n* = 5 biological replicate). (b) β‐Yariv gel diffusion assay of water soluble AGPs from Col‐0 and *CAGE1CAGE2* lines extracted from the first 10 cm of inflorescence stem. Numbers 1, 2 and 3 indicate biological replicates. Equal loading based on stem dry weight. (c) Agarose gel electrophoresis and β‐Yariv staining of water soluble AGPs from Col‐0 and *CAGE1CAGE2* lines extracted from the first 10 cm of inflorescence stem. 20 μg of Gum Arabic was loaded as a control. [Colour figure can be viewed at wileyonlinelibrary.com]

CAGE1 and CAGE2 are putative β1,3‐galactosyltransferases with a proposed role in AGP glycan biosynthesis (Egelund et al., 2018; Qu et al., 2008). To investigate this possibility, we measured the amounts of AGP linked β1,3‐galactan in *cage1cage2* and WT lines using β‐Yariv phenylglycoside. β‐Yariv binds specifically to β1,3‐galactan consisting of a minimum of seven monomeric units, and can be used to quantify AGP galactans in plant extracts (Kitazawa et al., 2013). β‐Yariv precipitation, followed by spectrophotometric quantification, revealed that, compared to WT, the *cage1cage2* lines contained approximately 35%, 22% and 25% less AGPs in the inflorescence stem, 7‐day‐old seedlings and 4‐day‐old etiolated hypocotyls, respectively (Figure 6a). The inflorescence stems were used for further AGP characterization. Soluble AGPs were extracted from the stems with hot water and analyzed using a β‐Yariv gel diffusion assay and gel electrophoresis. The gel diffusion assay confirmed the reduction in β‐Yariv accessible β‐1,3 galactan in *cage1cage2* relative to WT levels (Figure 6b), whereas the agarose gel electrophoresis revealed no major changes in the molecular weight distribution of AGPs (Figure 6c). However, it is possible that in *cage1cage2* a reduction or absence of β‐1,3 galactan on CAGE1/2 targets prevents their β‐Yariv precipitation in which case they would not appear in the electrophoresis assay. These results, combined with the CAGE1/2‐YFP Golgi localisation and their predicted β1,3‐galactosyltransferase domain, lead us to hypothesize that CAGE1 and CAGE2 synthesize glycans on AGPs or related glycoproteins participating in cellulose biosynthesis.

## DISCUSSION

We identified two genes from the GT31 family, here named *CAGE1* and *CAGE2*, based on the co‐expression of recently characterized Arabidopsis exo‐β1,3‐galactosidase genes (Nibbering et al., 2020). Subcellular localisation of CAGE1‐ and CAGE2‐YFP fusions established that they are located in the *cis*‐Golgi and/or medial‐Golgi, and characterization and complementation of *cage1cage2* mutants showed that the proteins act redundantly during both primary and secondary cell wall biosynthesis. *cage1cage2* mutants showed stunted seedling growth, cell expansion defects in etiolated hypocotyls and shortened inflorescence stems. When compared with WT plants, the *cage1cage2* mutants showed thinner and weaker (as evidenced by xylem vessel collapse) stem xylem fiber and vessel cell walls. The seedling and etiolated hypocotyl defects are similar to that previously observed in mutants with impaired primary cell wall cellulose biosynthesis (Persson et al., 2007), whereas the thinner xylem cell walls in the stem, along with the collapsed xylem vessels, are characteristic of secondary cell wall cellulose biosynthesis defects (Taylor et al., 2003; Turner & Somerville, 1997). These cell wall defects, when considered together with the reduced cellulose content in both *cage1cage2* seedlings and stems and the increased sensitivity to the cellulose biosynthesis inhibitor isoxaben of both the single and double mutants, indicate that CAGE1 and CAGE2 participate in cellulose biosynthesis. Observations of decreased secondary cell wall CESA levels in inflorescence stems and reduced cellulose biosynthesis rates in *cage1cage2* seedlings further support this hypothesis. The reduction in cellulose biosynthesis is unlikely to be triggered by a signaling cascade that regulates *CESA* transcription because the *cage1cage2* mutants did not show reduced primary or secondary cell wall *CESA* transcript levels.

The role of CAGE1/2 in cellulose biosynthesis may be indirect because the reduced β‐Yariv binding in *cage1cage2* mutants and the Golgi localization of both CAGE1‐YFP and CAGE2‐YFP suggest that these proteins are involved in the synthesis of AGP glycans. The simultaneous reduction in cellulose and AGPs observed in *cage1cage2* mutants raises the possibility that CAGE1 and CAGE2 act on a specific subgroup of AGPs that are involved in cellulose biosynthesis. One possibility is the FLA subgroup of AGPs, which have been linked to cellulose biosynthesis (MacMillan et al., 2010; Basu et al., 2016; Wang et al., 2015a; Ma et al., 2022. In this context, it is worth noting that even a 90% decrease in β‐Yariv precipitable AGPs in the hydroxyproline galactosyltransferase mutant *hpgt1,2,3* only had a slightly negative effect on the growth of Arabidopsis seedlings or young rosette‐stage plants (Ogawa‐Ohnishi & Matsubayashi, 2015). In comparison, the approximately 22–25% reduction in β‐Yariv binding measured in *cage1cage2* seedlings correlated with strong cell expansion defects and reduced root growth. Furthermore, Zhang et al. (2021) recently characterized a quintuple hydroxyproline galactosyltransferase mutant *galt2galt3galt4galt5galt6*, which exhibited an approximately 80% decrease in β‐Yariv precipitable AGPs in rosette leaves and a 50–60% decrease in the stems and siliques. The quintuple mutant showed reduced overall growth, impaired root growth, abnormal pollen, shorter siliques and reduced seed set, although the phenotype is still relatively mild compared to the strong growth defects observed in the *cage1cage2* mutants. This supports the hypothesis that the involvement of CAGE1 and CAGE2 in cellulose biosynthesis is linked to a specific glycosylation process rather than AGP glycosylation in general. Based on similar reasoning, it is also unlikely that CAGE1/2 would be involved in *N*‐glycosylation because mutants lacking GALT1 (i.e. a β1,3‐galactosyl transferase essential for adding a β1,3‐galactose to *N*‐glycans) did not display obvious phenotypic differences compared to WT plants (Strasser et al., 2007).

It is possible that CAGE1 and CAGE2 synthesize glycans directly attached to CESAs or other proteins involved in cellulose biosynthesis. The possibility of CESA *N*‐glycosylation has been investigated before, but considered unlikely because no molecular weight differences were observed between the CESAs from the PNGase‐treated protein extract of the *N*‐glycosylation deficient mutant *cgl1* and the CESAs from untreated WT plants (Gillmor et al., 2002). In the present study, we did not observe molecular weight shifts in the PAGE electrophoresis of secondary cell wall CESAs that would support differential glycosylation in the *cage1cage2* background (Figure S11). Of the proteins known to participate in cellulose biosynthesis, the plasma membrane‐associated, GPI‐anchored COBRA (COB) protein, and KOR1 have been shown to be *N*‐glycosylated (Roudier et al., 2005; von Schaewen et al., 2015). COB and KOR1 do not contain AGP‐like *O‐*glycosylation motifs, but their AG glycosylation cannot be excluded. Members of the COBRA‐LIKE (COBL) family have also been associated with cellulose biosynthesis in Arabidopsis (Ben‐Tov et al., 2015; Brown et al., 2005) eucalyptus (Thumma et al., 2009) and rice (Dai et al., 2011; Liu et al., 2013), and some of the COBLs contain predicted AG sites (Borner et al., 2003). However, the presence of AGs in COBLs has not been experimentally validated. Furthermore, AGP *O‐*glycosylation motifs are not fully understood, as shown by the presence of AGP glycans on the mutant form of FLA4, which lacks all of the predicted AGP glycosylation motifs (Xue et al., 2017).

Interestingly, a reduction in cellulose biosynthesis was also observed in mutants lacking the Golgi‐localized STELLO proteins from the GT75 family (Zhang et al., 2016). STELLO proteins interacted with CESAs in yeast two‐hybrid experiments and bimolecular fluorescence complementation assays in tobacco leaf epidermis cells (Zhang et al., 2016). The Golgi distribution, secretion and plasma membrane velocity of the CSC was impaired in the *stello* null mutant, which translated to reduced cellulose biosynthesis rates. The GT75 domain was shown to be essential for STELLO function, but the enzymatic activity of these proteins has not yet been established. It was hypothesized that STELLO may glycosylate CSCs or proteins that are associated with the CSCs and thereby affect CSC function and/or localization (Zhang et al., 2016).

Based on our results, we hypothesize that a defect in the glycosylation of cellulose biosynthesis associated proteins in *cage1cage2* mutant is linked to reduced CESA protein levels, which manifests as decreased cellulose content and a decrease in β‐Yariv accessible AGPs. Both CAGE1 and CAGE2 contain a domain of unknown function (DUF4094) in their N‐terminus (Figure S12). The GT31 family enzymes from *Homo sapiens* contain a N‐terminal galectin domain involved in substrate recognition (Barondes et al., 1994; Dodd & Drickamer, 2001). The galectin domain is an evolutionarily conserved carbohydrate recognition domain that can bind β‐galactosides. However, in Arabidopsis clade I–III GT31s, the galectin domain has been replaced by the DUF4094 (Qu et al., 2008). We speculate that this domain could be involved in recognizing targets or specific glycosylation structures, and guide the activity of CAGE1/CAGE2 on specific targets. However, further experimental work will be necessary to establish the enzymatic activity of CAGE1/2, as well as whether a specific AGP subgroup and/or other glycoproteins comprise their direct targets *in vivo*. At this point, the results open a new line of investigation into a Golgi localized CAGE1/2 process with significant repercussions on cellulose biosynthesis.

## EXPERIMENTAL PROCEDURES

### Plant material and growth conditions

The Arabidopsis (*Arabidopsis thaliana*) ecotype Columbia (Col‐0) was used as a WT control. The following Arabidopsis T‐DNA insertion lines (Col‐0) were ordered from the Nottingham Arabidopsis Stock Centre (Nottingham, UK): *cage2‐1* (SALK_110901); *cage2‐2* (SALK_150336); *cage1‐1* (SALK_069854); *cage1‐2* (SALK_053895); CESA4 null mutant (SALK_084627; *irx5‐4*); CESA7 null mutant (GABI_819B03; *irx3‐7*); and CESA8 null mutant (GABI_339E12; *irx1‐7*). The T‐DNA location of the *CAGE* lines was determined by a PCR and sequencing using gene‐ and T‐DNA specific primers (Table S2). Plants were grown on soil under a 16:8 h photocycle at 22 °C of 16 h light and 65% relative humidity. The plants were exposed to a light intensity of 150 μmol m^−2^ sec^−1^, which was emitted by a NS12 light emitting diode (LED) tubes (Valoya Oy, Helsinki, Finland).

### Plant vector construction and transformation

The genomic sequences of *CAGE1‐YFP* and *CAGE2‐YFP* were amplified using primers listed in Table S2. The amplified DNA fragments were cloned into the pDONR207 plasmid via Gibson assembly and recombined into pHGY (Kubo et al., 2005). Stable transgenic *cage1‐1cage2‐1* plants carrying either pCAGE1:*CAGE1‐YFP* or pCAGE2:*CAGE2‐YFP* were generated by *Agrobacterium tumefaciens*‐mediated transformation, as described in more detail by Nibbering et al. (2020). Transgenic seedlings were selected by hypocotyl elongation on ½ MS plates supplemented with 30 μg ml^−1^ hygromycin.

### Hypocotyl elongation and isoxaben treatment experiments

Seeds were surface‐sterilized with 70% ethanol and stratified for 3 days on ½ MS agar plates (0.9% agar, 5 mm MES, pH 5.7). For isoxaben treatment, medium was supplemented with freshly prepared isoxaben to a final concentration of 0.5 and 1 nm. The seeds were exposed to light for 6 h and then placed in the dark for 4 days. The plates were scanned and hypocotyl elongation was quantified with imagej (NIH, Bethesda, MD, USA). Graphs and statistics on isoxaben experiment were performed using prism (GraphPad Software Inc., San Diego, CA, USA).

### Light microscopy and TEM

For light microscopy, the bottom 1 cm of 10‐week‐old inflorescence stems was cut into 70‐μm cross‐sections with a vibratome. The cross‐sections were stained for 1 min in 0.01% toluidine blue and washed twice with water. Images of the cross‐sections were captured using an Axioplan microscope (Carl Zeiss, Oberkochen, Germany) with a camera attachment.

For the TEM, the bottom 1 cm of 10‐week‐old inflorescence stems was cut into small pieces and fixed in 2.5% glutaraldehyde (TAAB Laboratories, Aldermaston, UK) and 4% paraformaldehyde in 0.1 m sodium cacodylate buffer, and then further post‐fixed in 1% aqueous osmium tetroxide (TAAB Laboratories). Samples were dehydrated in ethanol and propylene oxide and finally embedded in Spurr's resin (TAAB Laboratories). All of the samples were processed using a Pelco Biowave Pro+ microwave tissue processor (Ted Pella, Redding, CA, USA). The 70‐nm ultrathin sections were post‐contrasted in uranyl acetate and Reynolds' lead citrate and further examined with a Talos L120C (FEI, Eindhoven, The Netherlands) operating at 120 kV. Micrographs were acquired with a Ceta 16M CCD camera (FEI) using tem image & analysis, version 4.17 (FEI).

### Quantification of the interfascicular fiber cell wall thickness

The interfascicular fiber cell wall thickness was measured from the TEM images of stem cross‐sections using imagej. The cell wall thickness was calculated by dividing the total cell wall area of an interfascicular fiber cell by the cell perimeter length.

### Wet chemical analysis of cell walls

The bottom 10 cm of 10‐week‐old soil‐grown plants was lyophilized and ground with a ball mill. The alcohol insoluble residue (AIR1) and starch (AIR2) were removed in accordance with the protocol described by Nibbering et al. (2020). Crystalline cellulose was quantified according to the methodology described by Updegraff (1969). Monosaccharide composition was determined as described by Latha Gandla et al. (2015), whereas lignin content was quantified with a lignin standard in accordance with the protocol described by Fukushima & Hatfield (2004).

### AGP analysis using β‐Yariv precipitation

β‐Yariv was synthesized as descibed by Yariv et al. (1962). The first 10 cm of inflorescence stems, 4‐day‐old hypocotyls and 7‐day‐old seedlings was harvested, lyophilized and ball milled. The amount of AGPs in 1.5 mg of dry weight sample was quantified in accordance with the protocol described by Lamport (2013) via a gum arabic standard. β‐Yariv gel diffusion and electrophoresis was performed as described by Veenhof & Popper (2020). Briefly, soluble AGPs were extracted by adding 50 μl of demineralized water per mg of dry weight. The samples were incubated for 30 min at 50°C and vortexed every 10 min. The samples were spun down at full speed for 2 min and the supernatant was transferred to a new tube. The supernatants were concentrated using a SpeedVac (Thermo Fisher Scientific, altham, MA, USA) until a desired volume was reached. For β‐Yariv gel diffusion, soluble AGPs from a 3‐mg dry weight sample were loaded on the gel. For β‐Yariv gel electrophoresis, soluble AGPs from a 10‐mg dry weight sample were loaded on the gel.

### Visualization of co‐localization using confocal microscopy

The lines *pCAGE1:CAGE1‐YFP* and *pCAGE2:CAGE2‐YFP* co‐expressing the marker lines SYP32‐mCherry or SYP43‐mCherry were generated by plant crossing, and imaged in accordance with the methodology provided in Nibbering et al. (2020).

### *CESA* expression, RT‐PCR and CESA western blot analysis

Four‐day‐old dark‐grown hypocotyls and the first 10 cm of 20‐cm inflorescent stems were flash‐frozen in liquid nitrogen and ground with a mortar and pestle. Total RNA was extracted using Trizol RNA Isolation Reagent (Thermo Fisher Scientific) (Simms et al., 1993). Any potentially contaminating DNA was removed with DNase treatment, after which cDNA was synthesized using the Maxima First Strand cDNA Synthesis Kit (Thermo Fisher Scientific). CESA expression was measured using *CESA* and *UBQ1* specific primers as described by Wang et al. (2015b).

Proteins were extracted from a 30‐mg fresh weight sample based on the methodology reported by Hill et al. (2014). In total, 10 μg of protein was loaded into every well of the SDS‐PAGE gel. Following electrophoresis, the proteins were transferred to a polyvinylidene fluoride membrane. The membranes were blocked in 5% milk powder in Tris‐buffered saline‐Tween 20 (TBST). The membranes were blotted with the primary antibodies CESA4 (AS12 2582; Agrisera, Vaesterbotten, Sweden), CESA7 (AS12 2581; Agrisera) or CESA8 (AS12 2580; Agrisera), prepared as 1:1000 dilutions in 2.5% milk powder in TBST. The membranes were then blotted with the goat anti‐rabbit IgG antibody (H+L), peroxidase PI‐1000 (vector), prepared as a 1:10000 dilution in 2.5% milk powder in TBST.

### ^13^C labelling of Arabidopsis seedlings

Seeds (20 mg) were surface‐sterilized for 5 min in 70% ethanol and 10 min in chlorine solution, and then washed three times for 5 min in sterile demineralized water and stratified for 3 days at 4°C. The seedlings were grown for 8 days in 50 ml of ½ MS media (5 mm MES, 1% glucose, pH 5.7) in 250‐ml flasks under a 16:8 h photocycle (light, 24°C; dark, 18°C). On the day 8, 1% glucose (90% glucose, 10% ^13^C‐glucose, CAS number: 201136‐45‐0; Sigma‐Aldrich, St Louis, MO, USA) was added to the seedlings and samples were taken 0, 8, 16 and 24 h after the addition of glucose. The seedlings were washed twice in demineralized water and flash‐frozen in liquid nitrogen. The seedlings were lyophilized and ground with a ball mill (total dry weight). The AIR1/AIR2 fractions were removed from the dry cell wall powder and the crystalline cellulose was extracted according to the protocol reported by Updegraff (1969). From the total dry weight and crystalline cellulose, 5 mg was weighed down, packed and measured with an elemental analyzer (Flash EA 2000; Thermo Fisher Scientific) coupled to an isotope ratio mass spectrometer (DeltaV; Thermo Fisher Scientific) to determine the ^13^C content (Werner et al., 1999). The ^13^C fraction of total carbon was calculated from working standards of wheat and maize flours calibrated against IAEA‐600, IAEA‐CH‐6 and USGS40 reference standards.

### Targeted CESA proteomics

Unique peptides were selected as quantitative surrogates for CESA4 (AT5G44030, APEFYFSEK), CESA7 (AT5G17420, ATDDDDFGELYAFK) and CESA8 (AT4G18780, DIEGNELPR) and analyzed as described in Zhang et al. (2018). An isotope labeled peptide for CESA4 (FDGID^**13**^ **L**ANDR) was also used in the analysis. The first 10 cm of a 20‐cm inflorescent stem was flash‐frozen in liquid nitrogen and ground with a mortar and pestle. Proteins were extracted and trypsin‐digested as described by Zhang et al. (2018), followed by a post digestion clean‐up on an Oasis® HLB μElution plate (Waters Corp., Milford, MA, USA). Approximately 0.5 μg of each sample were loaded. For liquid chromatography‐multiple reaction monitoring (MRM), samples were analyzed by reversed‐phase liquid chromatography‐electrospray ionization‐MS/MS using a nanoACQUITYTM UPLC (Waters Corp.) system connected to a triple‐quadrupole mass spectrometer (Xevo TQ‐S; Waters Corp.) in trapping mode. The trapping flow rate was 5 μl min^−1^ and the trapping time was 3 min. The separation cycle time took 32 min at a flow rate of 2 μl min^−1^. Peptides were loaded onto a BEH iKey Separation Device (C18, 1.8 μm, 150 μm innber diameter × 50 mm; Waters Corp.) in 99% solvent A (0.1% formic acid in H_2_O) and 1% of solvent B (0.1% formic acid in acetonitrile). The percentage of solvent B was elevated from 1% to 30% in an 18‐min linear gradient, then increased from 30% to 40% solvent B in 2 min, followed with a 2.5‐min linear gradient to 95% of B and held at 95% for 2.5 min. After that, the column was balanced by ramping to 1% solvent B in 0.5 min and flushing for 7 min. Data acquisition was performed using the parameters: capillary voltage, 3.2 kV; source temperature, 120°C; cone gas, 60 L h^−1^. Five transitions per peptide were monitored in a scheduled way, with a cycle time of 2 sec and a 4‐min MRM detection window. The cone voltage and collision energy for each transition were performed as described by Zhang et al. (2018). Data were imported into skyline, version 4.1.0 (MacCoss Lab Software; Washington University, Seattke, WA, USA) with Savitzky–Golay smoothing. All peaks were manually inspected to ensure correct peak detection and accurate integration.

## CONFLICT OF INTEREST

The authors declare no conflict of interest.

## AUTHOR CONTRIBUTIONS

PN, RC and GW performed experiments and analyzed data. PN and TN planned experiments and analyzed data. PN and TN wrote the manuscript with help from the other authors.

## DATA AVAILABILITY

All relevant data can be found within the manuscript and its supporting materials.

## Supporting information


**Figure S1.** Phylogenetic tree of GT31 family linked to β1,3‐galactosyltransferase activity.
**Figure S2.** RT‐PCR analysis of CAGE transcripts.
**Figure S3.** Light micrographs of stem cross‐sections.
**Figure S4.** Light micrographs of stem cross‐sections.
**Figure S5.** Electron micrographs of xylem fibers and vessels.
**Figure S6.** Interfascicular fiber wall thickness.
**Figure S7.** Etiolated hypocotyl length of CAGE‐YFP lines.
**Figure S8.** Complementation of *cage1cage2* with *CAGE‐YFP* constructs.
**Figure S9.** Cellulose content in the complemented *cage1cage2* lines.
**Figure S10.** Stem lignin content in wild‐type and *cage* mutants.
**Figure S11.** Western blot of CESA4, 7 and 8 in wild‐type and *cage1cage2*.
**Figure S12.** Amino acid sequence alignment of CAGE1 and CAGE2.
**Table S1.** GH43 co‐expressed genes.
**Table S2.** Primers used in the present study.

**Appendix** **S1.** Supporting information.
