# Supplementary material for: CAGEs are Golgi‐localized GT31 enzymes involved in cellulose biosynthesis in Arabidopsis
Source: Plant J. 2022 Mar 31;110(5):1271–85. doi: 10.1111/tpj.15734 (PMC9321575; doi:10.1111/tpj.15734)
Supplement: Supplementary file 1 — Figure S1. Phylogenetic tree of GT31 family linked to β1,3‐galactosyltransferase activity. Figure S2. RT‐PCR analysis of CAGE transcripts. Figure S3. Light micrographs of stem cross‐sections. Figure S4. Light micrographs of stem cross‐sections. Figure S5. Electron micrographs of xylem fibers and vessels. Figure S6. Interfascicular fiber wall thickness. Figure S7. Etiolated hypocotyl length of CAGE‐YFP lines. Figure S8. Complementation of cage1cage2 with CAGE‐YFP constructs. Figure S9. Cellulose content in the complemented cage1cage2 lines. Figure S10. Stem lignin content in wild‐type and cage mutants. Figure S11. Western blot of CESA4, 7 and 8 in wild‐type and cage1cage2. Figure S12. Amino acid sequence alignment of CAGE1 and CAGE2. Table S1. GH43 co‐expressed genes. Table S2. Primers used in the present study. [file TPJ-110-1271-s001.pdf]

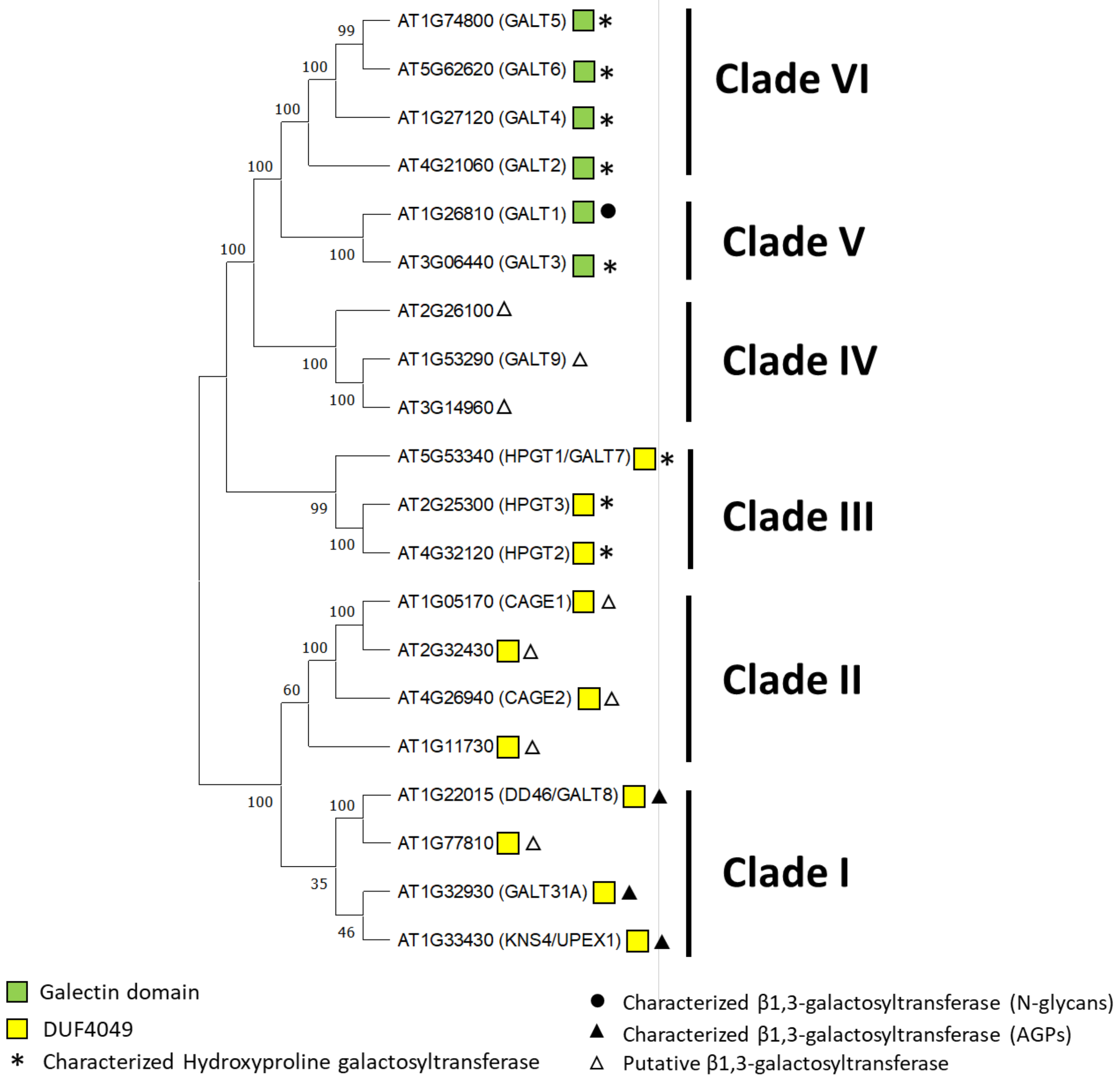

Supplementary figure 1: Phylogenetic tree of the *Arabidopsis thaliana* GT31 family clade I-VI based on Qu et al., 2008. The amino acid sequences of *Arabidopsis thaliana* GT31 family members with (putative) roles in AGP glycan synthesis based on Qu et al., (2008). The amino acid sequences were aligned via ClustalW. Phylogenic trees were constructed using minimum-evolution method of MEGA-X in default mode with bootstrap test of 1000 replicates. The numbers beside the branches correspond to % bootstrap values.

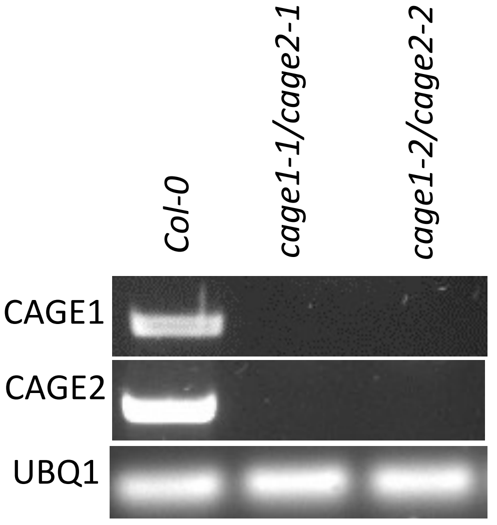

Supplementary figure 2: RT-PCR analysis of transcripts from the first 10 cm of 20 cm long inflorescence stems from *Col-0, cage1-1/cage2-1* and *cage1-2/cage2-2.*

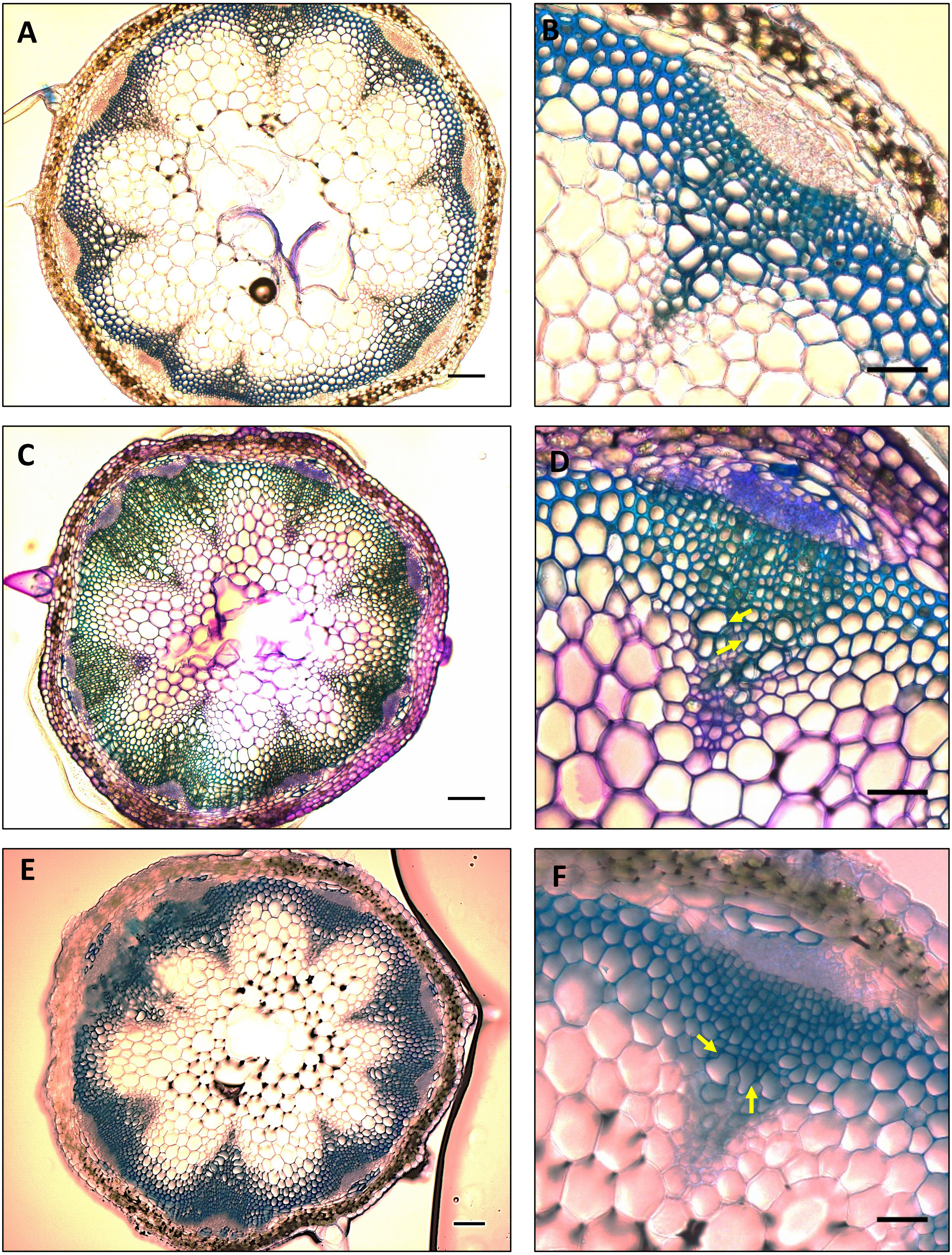

Supplementary figure 3: Cross sections of 10-week-old inflorescence stem from *Col-0* and *cage1cage2* lines stained with toluidine blue. *Col-0* (A, B), *cage1-1/cage2-1* (C, D) and *cage1-2/cage2-2* (E, F). The yellow arrows indicate collapsed xylem vessels. Scale bar A,C,E = 100 µm and scale bar B,D,F = 50 µm.

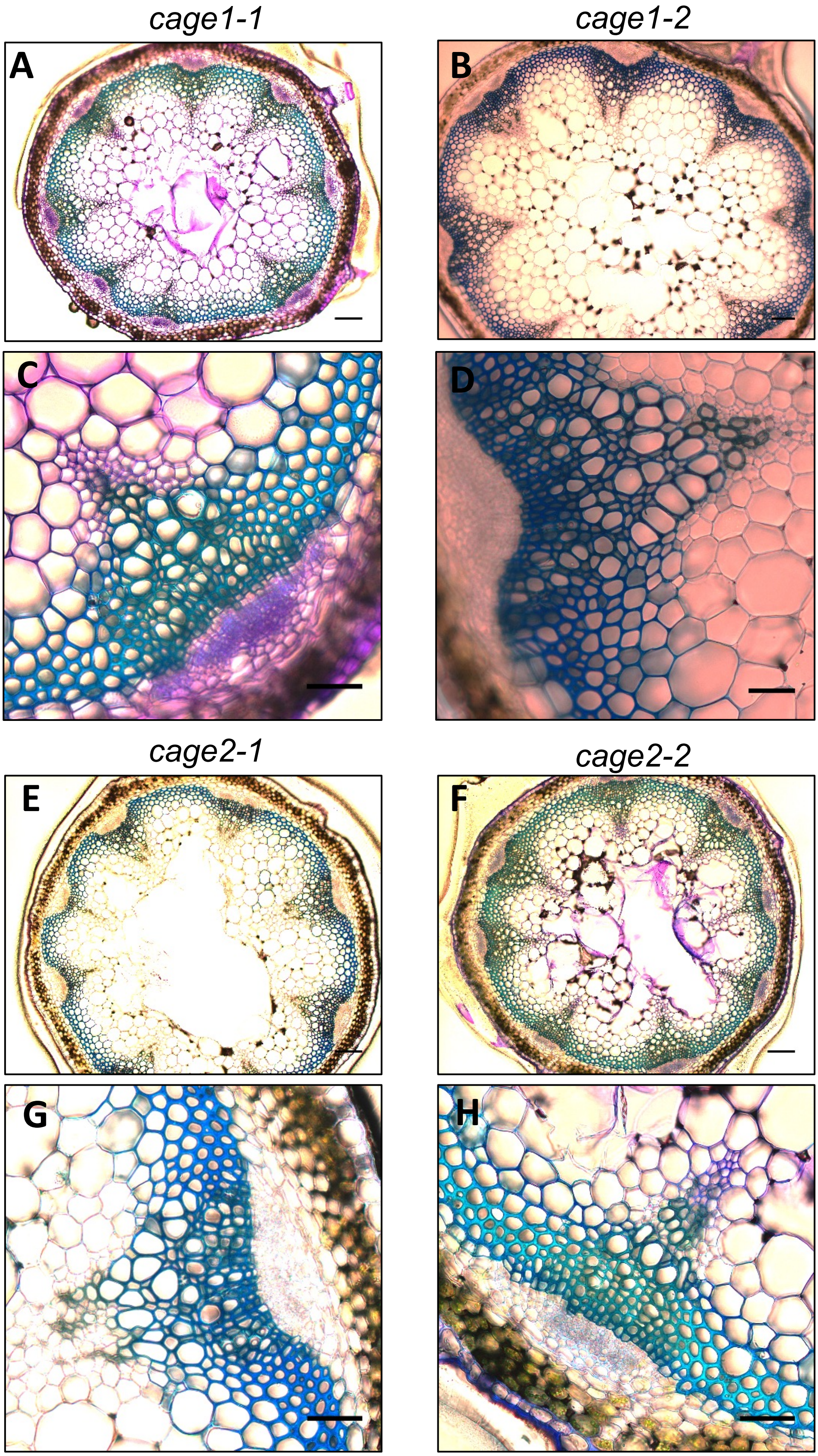

Supplementary figure 4: Cross sections of 10-week-old inflorescence stems from *cage1* and *cage2* lines stained with toluidine blue. *cage1-1* (A, C), *cage1-2* (B, D), *cage2-1* (E,G), *cage2-2* (F,H). Scale bar A,B,E,F = 100 µm and scale bar C,D,G,H = 50 µm.

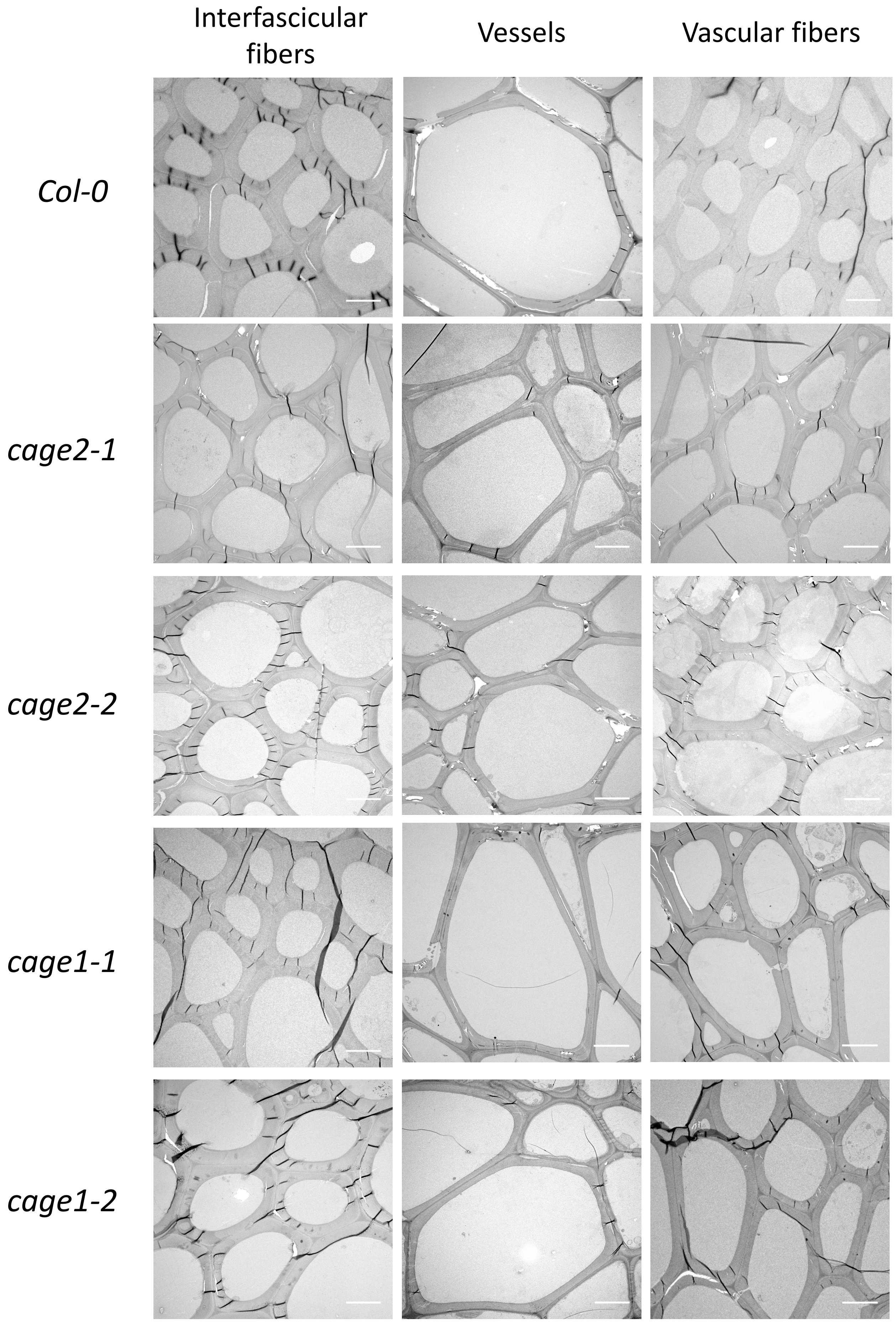

Supplementary figure 5: Transmission Electron Microscope (TEM) images of transverse sections of xylem fibers and vessels of *Col-0, cage2-1, cage2-2, cage1-1 and cage1-2.* Scale bar = 5 µm

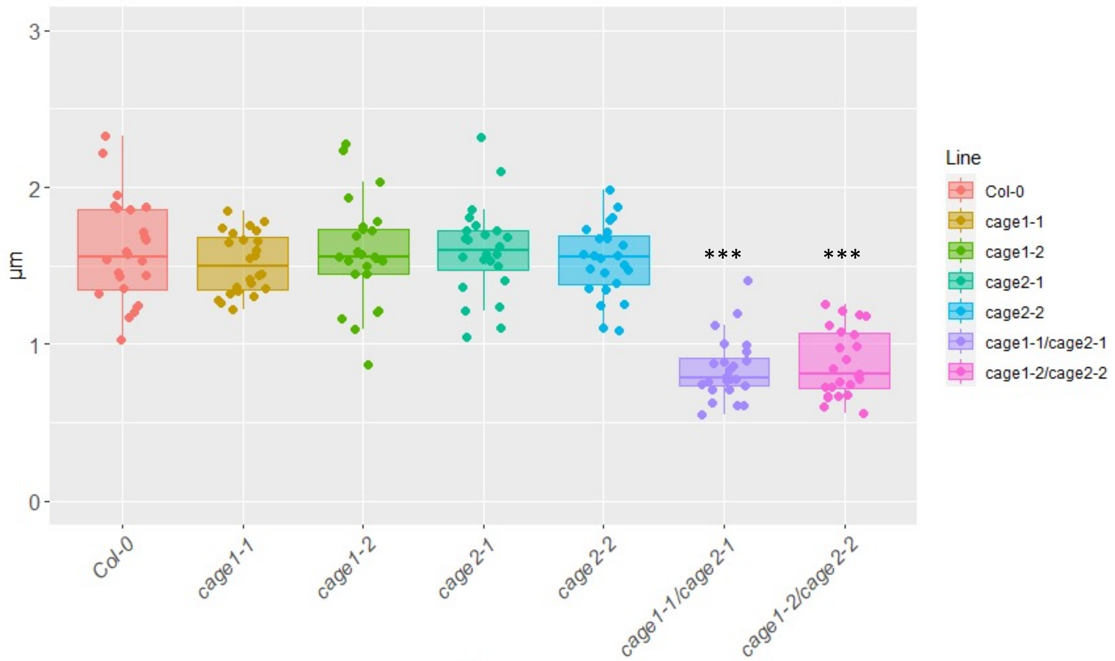

Supplementary figure 6: Interfascicular fiber thickness of *Col-0* and the *cage* lines

In the boxplot, the middle horizontal line represents the median, the two boxes denote the 25th and 75th percentiles, the whiskers denote the 1.5 interquartile range limits. The dots represent the individual measurements (n= 4 biological replicates, 6 individual cells per biological replicate). Significance is calculated with a two-sided student's T-test. *** ≤0.001.

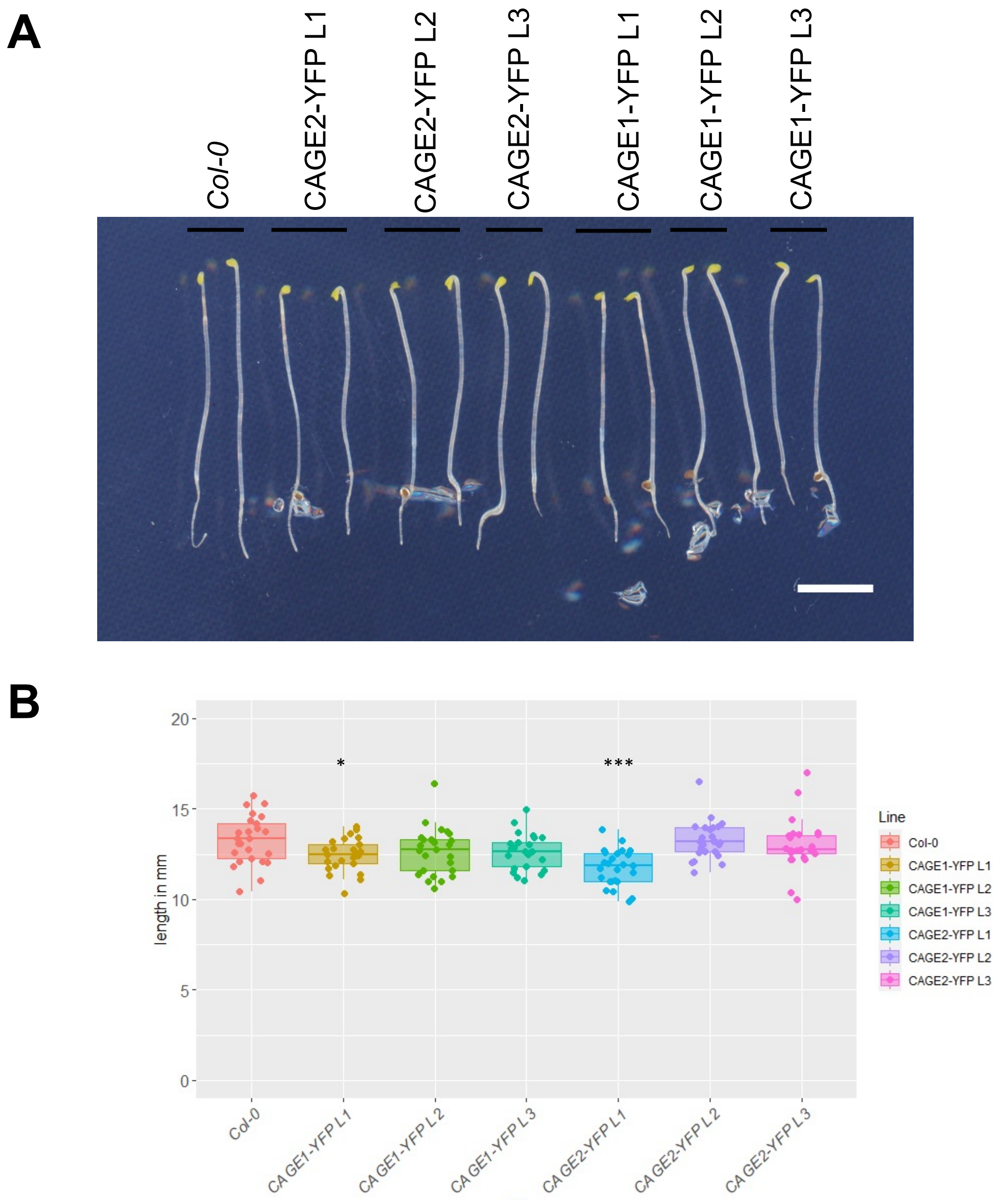

Supplementary figure 7: Length of four-day old etiolated Col-0 and CAGE1-YFP and CAGE2-YFP lines.
(A) Four-day old etiolated seedlings of Col-0 and *cage1-1cage2-1* lines expressing *cage1-YFP* or *cage2-YFP*. Scale bar = 5mm
(B) Hypocotyl length of 4-day old etiolated Col-0 and *cage1-1cage2-1* lines expressing *CAGE1-YFP* or *CAGE2-YFP*. In the boxplot, the middle horizontal line represents the median, the two boxes denote the 25th and 75th percentiles, the whiskers denote the 1.5 interquartile range limits. The dots represent the individual measurements (n = 25 biological replicates). Significance is calculated with a two-sided student's T-test. * ≤0.05, *** ≤0.001.

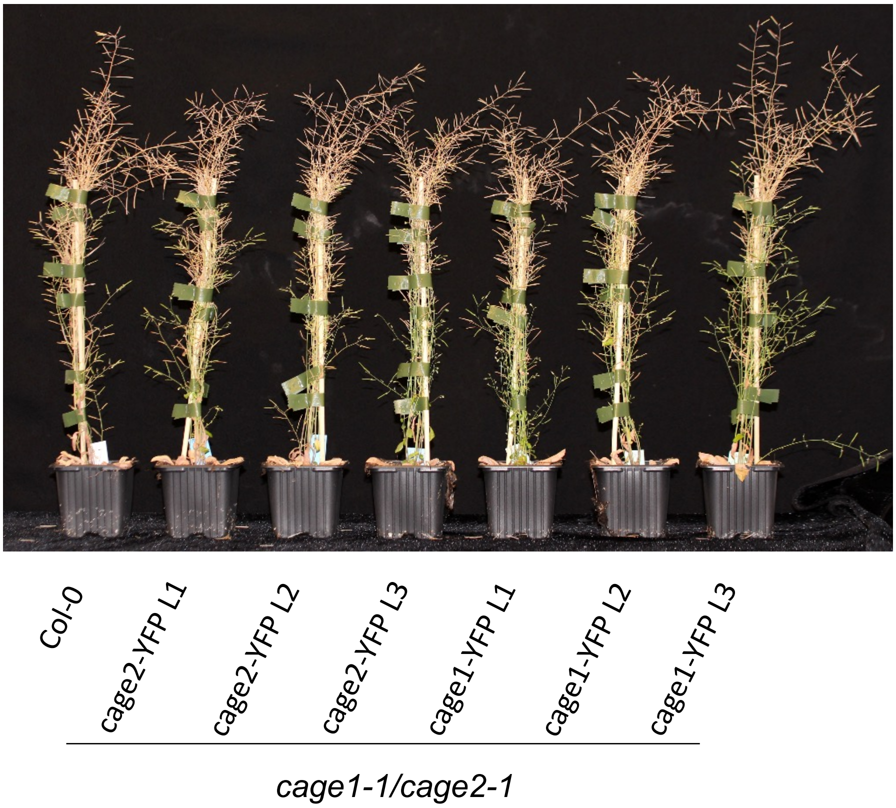

Supplementary figure 8: Ten-week-old Col-0 and *cage1-1cage2-1* plants transformed with *CAGE1-YFP* or *CAGE2-YFP* under their native promoters.

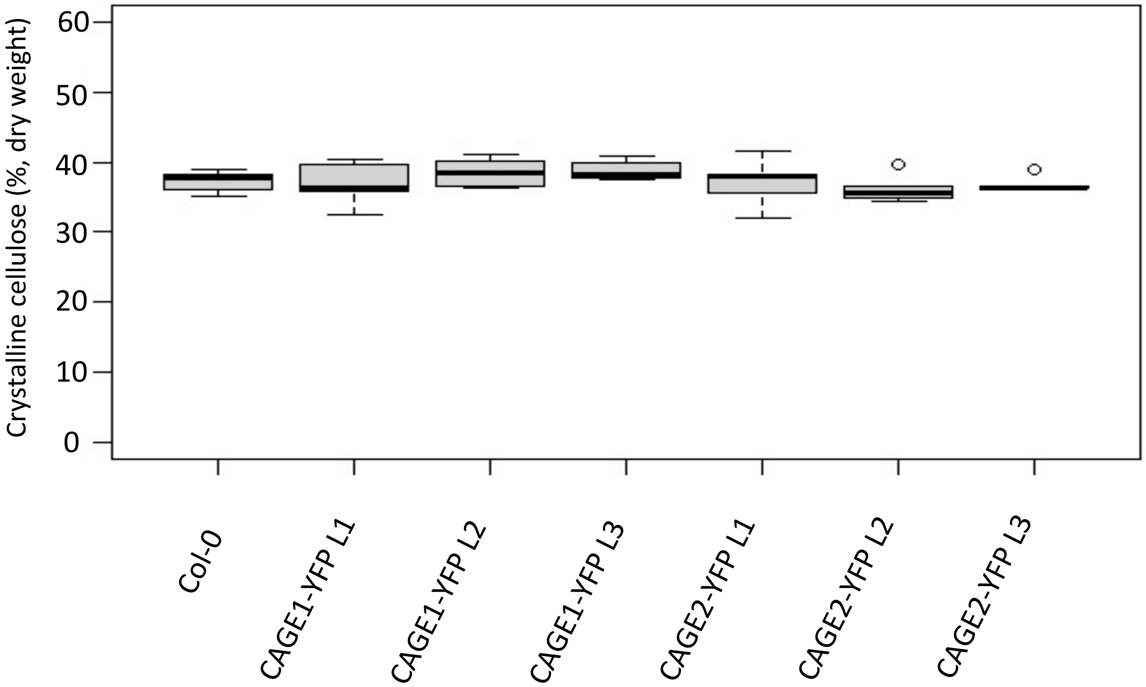

Supplementary figure 9: Crystalline cellulose content (% of dry weight) in 10-week-old Col-0 and *cage1-1cage2-1* lines expressing *CAGE1-YFP* or *CAGE2-YFP*.

In the boxplot, dark horizontal lines represent the median, the two grey boxes denote the 25th and 75th percentiles, the whiskers denote the 1.5 interquartile range limits, and the dots are outliers. (n = 6 biological replicates)

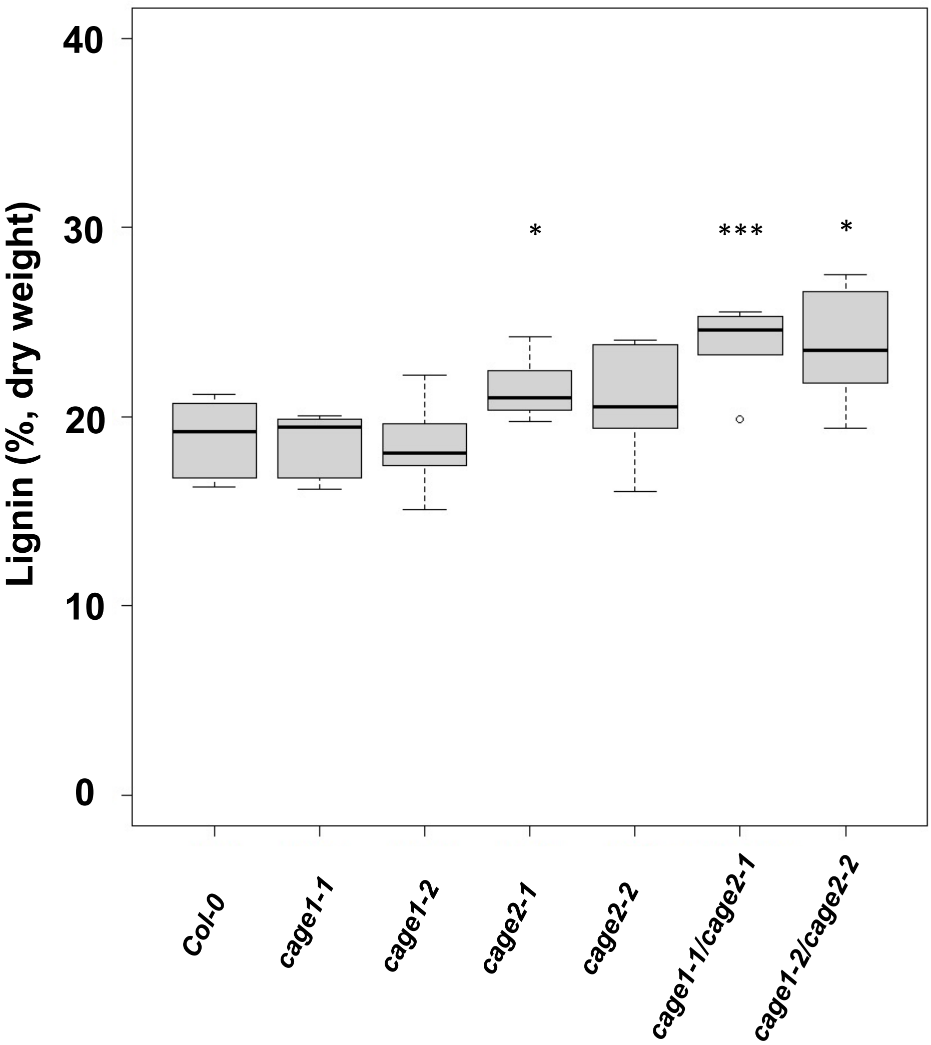

Supplemental figure 10: Lignin content (%, dry weight) in 10-week-old inflorescence stems of *Col-0, cage1, cage2* and *cage1cage2* lines.

Lignin content (% of dry weight) in 10-week-old inflorescence stems. In the boxplot, dark horizontal lines represent the median, the two grey boxes denote the 25th and 75th percentiles, the whiskers denote the 1.5 interquartile range limits, and the dots are outliers. *P < 0.05, ***P < 0.001 (unpaired t test, *n* = 6 biological replicates).

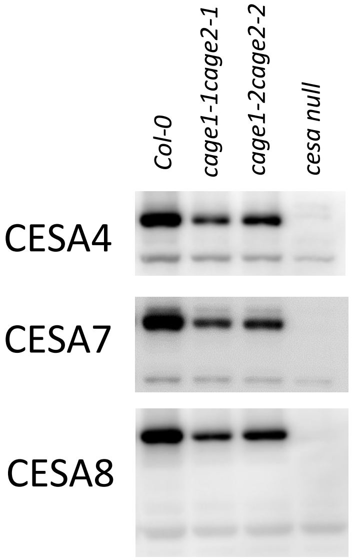

Supplementary figure 11: Western blot analysis using CESA4, 7 and 8 specific antibodies. 10ug of protein was loaded from from the bottom 10 cm of 20 cm inflorescence stems of *Col-0* and the *cage1cage2* plants. The *CESA null* controls correspond to the published lines *irx5-4* (*CESA4*), *irx3-7* (*CESA7*) and *irx 1-7* (*CESA 8*).

```
CAGE1   MSA----KIKGEYSSRSFVSRKWTILLCLGSFCVGMFFTNRMWNIPESKGMSHPSVTEAE      56
CAGE2   MSLKHHHRGLELSASKSFVSKKWTLFLCIGFFCAGILFSDRMWPEPESNVVSRDTVASDE      60
        **     :     :*:****:***::**:* **.*::*::***  ***: :*: :*:. *

CAGE1   RLKLVSEGCNPKAKEVKRDPQALFGEVANTHIALQTLDKTISSLEMELAAARSVQESLQN      116
CAGE2   RLRLESEDCDSSKKGLKRESKDILGDVYKSPDAIQTLDKTISKLETELADARAAQESIMN      120
        **:* **.*: . * :**: : ::*:* ::  *:********.** *** **:.***: *

CAGE1   GAPLSDDMGKKQPQEQRRFLMVVGINTAFSSRKRRDSIRATWMPQGEKRKRLEEEKGIII      176
CAGE2   GSPVSDDFKLPETVTKRKYLMVVGVNTAFSSRKRRDSVRATWMPPGEERKKLEEEKGIVM      180
        *:*:***:   :   :*::*****:************:****** **:**:*******::

CAGE1   RFVIGHSATTGGILDRAIEAEDRKHGDFLRLDHVEGYLELSGKTKTYFSTAFSMWDADFY      236
CAGE2   RFVIGHSSTPGGILDRAIQAEESKHGDFLRLDHVEGYLELSAKTKTYFTTAFAMWDADFY      240
        *******:* ********:**: ******************.******:***:*******

CAGE1   VKVDDDVHVNIATLGETLVRHRKKPRVYIGCMKSGPVLSQKGVRYHEPEYWKFGENGNKY      296
CAGE2   VKVDDDVHVNIATLGAELARYRMKPRVYIGCMKSGPVLAQKGVRYHEPEYWKFGEEGNKY      300
        ***************  *.*:* ***************:****************:****

CAGE1   FRHATGQLYAISRDLASYISINQHVLHKYANEDVSLGAWFIGIDVKHIDDRRLCCGTPPD      356
CAGE2   FRHATGQLYAISRELASYISINQNVLHKYVNEDVSLGSWFLGLDVEHVDDRRLCCGT-TD      359
        *************:*********:*****.*******:**:*:**:*:*********  *

CAGE1   CEWKAQAGNICVASFDWSCSGICRSADRIKEVHRRCGEGEKALWSATF*      404
CAGE2   CEWKAQAGNICVASFDWSCSGICRSADRMKDVHRRCGEGEKALLAASF*      407
        ****************************:*:************ :*:**
```

Supplementary figure 12: Amino acid sequence alignment of CAGE1 and CAGE2.
The amino acid sequences of CAGE1 and CAGE2 were aligned with ClustalW using standard settings . The sequence highlighted in red annotates the predicted membrane domain. The sequence highlighted in blue marks the predicted DUF4094 domain. The sequence highlighted in green marks the predicted galactosyltransferase domain.

**Supplementary table 2: Primers used in this study**		
**Genotyping primers**		
cage2-1 *cage2-2*	LP	TTTTCGAAACACCCAGATTTG
	RP	GAATTCCACCGGGAGTAGAAC
cage1-1	LP	AAGGCATGTCTCATCCATCTG
	RP	GTTTTCGTCTTGCCTGACAAC
cage1-2	LP	ATCTTTGAGCTGTTTTTGGGG
	RP	AATGTCTGCAGCAACTCTTGG
LB1.3	LB	ATTTTGCCGATTTCGGAAC
**RT-PCR**		
CAGE1_FW	ATGTCTGCGAAGATCAAAGG	
CAGE1_RV	TCAAAACGTGGCACTCCA	
CAGE2_FW	ATGTCGTTGAAGCATCATCA	
CAGE2_RV	TCAGAAAGATGCAGCCAG	
UBQ1_FW	TTCCTTGATGATGCTTGCTC	
UBQ1_RV	TTGACAGCTCTTGGGTGAAG	
**YFP constructs**		
CAGE2-YFP	LP	*AGAAAGCTGGGTCGGC***GAAAGATGCAGCCAGAAGGG**
	RP	*CAAAAAAGCAGGCTCC***ATGAAGCATGAGCTATGGGGA**
CAGE1-YFP	LP	AGAAAGCTGGGTCGGC**AAACGTGGCACTCCAAAGA**
	RP	CAAAAAAGCAGGCTCC**TCAGAAACCTGGTTGAGAAAA**
